# Co-inhibitory T cell receptor KLRG1: human cancer expression and efficacy of neutralization in murine cancer models

**DOI:** 10.18632/oncotarget.26659

**Published:** 2019-02-15

**Authors:** Steven A. Greenberg, Sek Won Kong, Evan Thompson, Stefano V. Gulla

**Affiliations:** ^1^ Department of Neurology, Brigham and Women's Hospital and Harvard Medical School, Boston, MA, USA; ^2^ Computational Health Informatics Program, Boston Children's Hospital, Boston, MA, USA; ^3^ Department of Pediatrics, Harvard Medical School, Boston, MA, USA; ^4^ Abcuro, Inc., Newton, MA, USA

**Keywords:** immune checkpoint receptor, immunotherapy, KLRG1, co-inhibitory receptor, murine cancer models

## Abstract

**Background:**

KLRG1 is a lymphocyte co-inhibitory, or immune checkpoint, receptor expressed predominantly on late-differentiated effector and effector memory CD8+ T and NK cells. Targeting of KLRG1 neutralization in murine cancer models has not previously been reported.

**Methods:**

We studied KLRG1 expression in human blood and tumor samples from available genomic datasets. Anti-KLRG1 neutralizing antibody was studied in the murine 4T1 breast cancer as monotherapy, and in the MC38 colon cancer and B16F10 melanoma models as combination therapy with anti-PD-1 antibody.

**Results:**

In human blood and tumor samples, KLRG1 expression is aligned with cytotoxic T and NK cell differentiation, and upregulated in human tumor samples after a variety of therapies, potentially contributing to adaptive resistance. In *in vivo* murine models, anti-KLRG1 antibody monotherapy in the 4T1 breast cancer model reduced lung metastases (decreased lung weights p=0.04; decreased nodule count p=0.002), while anti-KLRG1 + anti-PD-1 combination therapy in the MC38 colon cancer and B16F10 melanoma models produced synergistic benefit greater than anti-PD-1 alone for tumor volume (MC38 p=0.01; B16F10 p=0.007) and survival (MC38 p=0.02; B16F10 p=0.002).

**Conclusions:**

These studies provide the first evidence that inhibition of the KLRG1 pathway enhances immune control of cancer in murine models, and provide target validation for KLRG1 targeting of human cancer. The mechanism of efficacy of KLRG1 blockade in murine models remains to be determined.

## INTRODUCTION

Killer cell lectin-like receptor G1 (KLRG1) is a co-inhibitory, or immune checkpoint, receptor inhibiting the activity of T and NK cells. It's ligands are E-cadherin and N-cadherin with similar affinities of 7-12 μM [1], respective markers of epithelial and mesenchymal cells [2]. Whereas targeting of other co-inhibitory receptors for applications in oncology has gained widespread interest [3–5], including multiple FDA approvals for targeting CTLA-4, PD-1, and its ligand PD-L1, less attention has been focused on the therapeutic potential of KLRG1 modulation. Unlike the obvious enhanced immune activation present in CTLA-4 and PD-1 gene knockout mice [6, 7], KLRG1 knockout mice initially were found to have no abnormal features [8], though were subsequently found to have enhanced immunity in a tuberculosis challenge model [9]. No anti-mouse KLRG1 antibody with characterized neutralizing function has been available as a reagent for murine model studies, and no murine cancer studies targeting KLRG1 neutralization have been published. The characterization of KLRG1 as a “senescent” marker, but all other co-inhibitory receptors as “exhaustion” markers [10–12], may have contributed to relatively fewer studies of this molecule.

Nevertheless, previous studies have demonstrated that an anti-KLRG1 antibody can increase *ex vivo* human NK cell interferon-gamma secretion [13] and that anti-E-cadherin antibodies can result in enhanced *ex vivo* human CD8 T cell proliferation and NK cell cytotoxicity [14–16]. Because E-cadherin is also a ligand for the T cell receptor αEβ7 integrin, the effects of anti-E-cadherin antibodies leave uncertain the role of KLRG1 in human CD8 T cell activation. Here, we report on translational studies of human KLRG1 expression and the *in vivo* activity of an anti-mouse KLRG1 neutralizing antibody in murine cancer models.

## RESULTS

### KLRG1 is preferentially expressed on effector and effector memory CD8 T cells and NK cells and differentially expressed than PD-1

**Figure 1:**
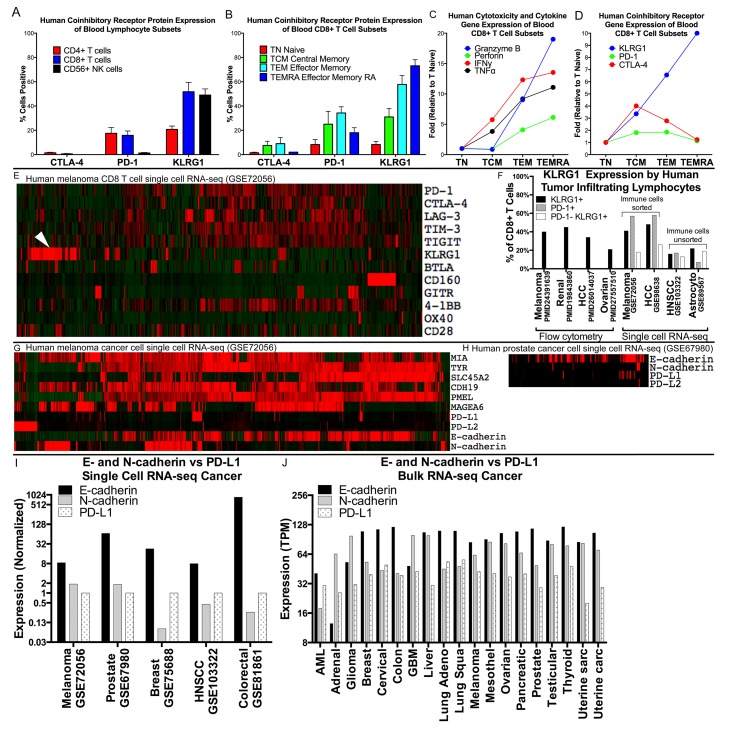
Expression of KLRG1 and its ligands in healthy blood and patient tumor samples **(A–D)** Expression of KLRG1 in healthy blood. (A) KLRG1 protein expression by flow cytometry is greater for CD8 T and NK cells than for CD4 T cells, distinct from CTLA-4 and PD-1, and (B) increases with CD8 T cell differentiation. (C–D) KLRG1 gene expression is aligned with cytotoxic potential of CD8+ T cells (e.g., granzyme B and perforin) **(E–F)** Expression of KLRG1 in tumor. (E) Co-inhibitory receptor gene expression in single cell RNA-seq human melanoma (GSE72046), in 1257 CD8+ T cells showing a distinct population of KLRG1+ cells (arrowhead) compared to PD-1, CTLA-4, LAG-3, TIM-3, and TIGIT. (F) KLRG1+ cells in human tumor infiltrating lymphocytes (TILS) from publications and datasets. **(G–J)** Expression of KLRG1 ligands in tumor. (G) Expression in 1184 melanoma cancer cells and (H) 177 prostate cancer cells showing many more KLRG1 ligand E- and N-cadherin positive cells than PD-1 ligand PD-L1 positive cells. (I) Multiple single cell RNA-seq cancer datasets showing E- or N-cadherin compared to PD-L1 expression (log-scale). (J) Bulk tumor RNA data from TCGA showing abundant E-cadherin expression compared to PD-L1 expression across 6,358 human cancer samples from 19 cancer types (log-scale).

We mined available gene expression datasets and publications (Supplementary Table 1) to compare human co-inhibitory receptor expression by various blood lymphocyte populations from healthy people. KLRG1 is differentially expressed from CTLA-4 and PD-1, with predominant expression on cytotoxic CD8 T and NK cells over CD4 T cells. Within the CD8+ T cell population, KLRG1 expression, unlike CTLA-4 and PD-1 expression, is linked to greater antigen-driven differentiation states, with increased expression on CD45RO+CCR7- T effector memory (TEM) and CD45RA+CCR7- T effector memory RA (TEMRA) cells compared to CD45RA+CCR7+ naïve T cells (TN) and CD45RO+CCR7+ central memory T cells (TCM) (Figure 1A, 1B). The cytotoxic potential of CD8+ T cells, as assessed by the presence of cytokine and cytotoxic molecules IFNγ, TNFα, perforin and granzyme B, is aligned with KLRG1, but not CTLA-4 or PD-1, expression (Figure 1C, 1D).

KLRG1 has been little studied in human tumor samples. Together with additional datasets containing single cell RNA-seq gene expression data from human cancer biopsies, KLRG1+ TILS accounted for 16-48% of CD8+ TILS, a frequency similar to that of PD-1+ TILS, in renal cell carcinoma, hepatocellular carcinoma, melanoma, ovarian cancer, HNSCC, and astrocytoma (Figure 1E, 1F). A distinct population of PD-1−KLRG1+ infiltrating CD8 T cells accounted for 13-26% of CD8+ TILS across a range of cancer types.

We also studied the expression of the KLRG1 ligands E-cadherin and N-cadherin in tumor sample data. Their transcripts were highly expressed in single cell RNA-seq data of melanoma, prostate, breast, HNSCC, and colorectal cancer cells with expression levels substantially higher than the PD-1 ligand PD-L1 (Figure 1G–1I). In bulk RNA data across 6,358 cancer samples from 19 different cancer types, E-cadherin and N-cadherin expression were similarly over-expressed compared to PD-L1 (Figure 1J).

### Inhibition of metastasis in the 4T1 breast cancer model with monotherapy

**Figure 2:**
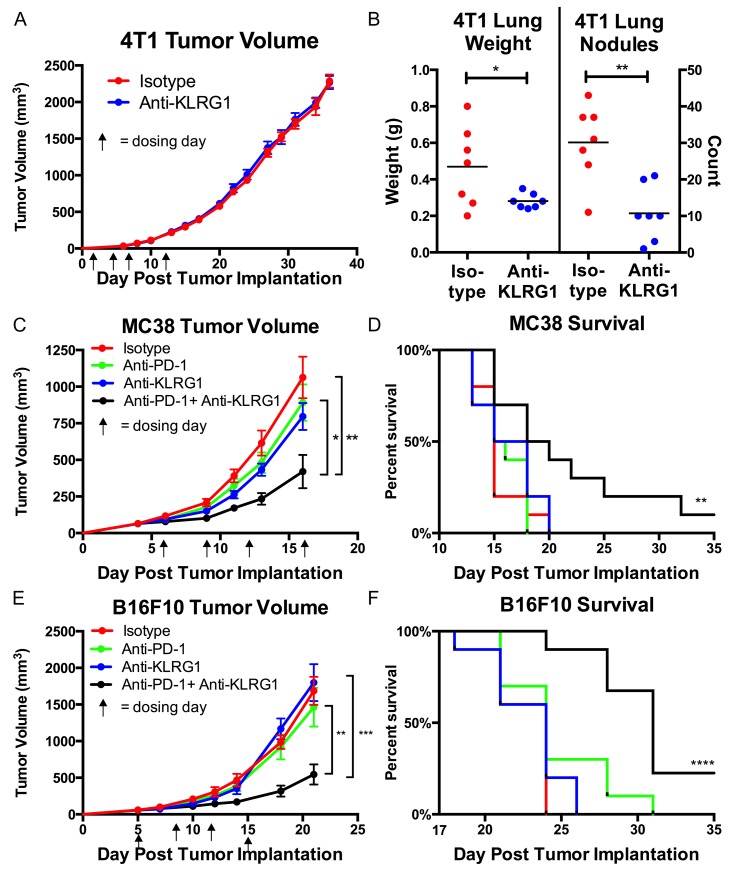
*In vivo* murine efficacy of anti-KLRG1 antibody **(A and B)** Inhibition of metastasis in 4T1 breast cancer model.(A) No effect on primary tumor growth. (B) Reduction in lung weights and nodule counts in anti-KLRG1 antibody treated mice. **(C–F)** Reduction in tumor growth and improvement in survival with anti-KLRG1 + anti-PD-1 combination therapy in (C, D) MC38 colon cancer model and (E, F) B16F10 melanoma model. Survival P-values compare combination therapy to control antibody. P-values: ^*^ <0.05, ^**^ <0.01, ^***^ <0.001, ^****^ <0.0001.

We confirmed that anti-KLRG1 antibody inhibited binding of mouse E-cadherin to KLRG1 (Supplementary Figure 1) and tested its effect on preventing metastasis in the 4T1 metastatic breast cancer model. 4T-1 cells express high levels of E-cadherin (Supplementary Figure 2). Although there was no effect of anti-KLRG1 antibody on primary tumor growth, anti-KLRG1 antibody significantly reduced lung metastases, measured by lung weight (p=0.04) and lung nodule count (p=0.002) (Figure 2A, 2B).

### Inhibition of primary tumor growth and improved survival in the MC38 colon cancer and B16F10 models with combination therapy

We tested anti-KLRG1 antibody as a combination therapy with anti-PD-1 antibody (Figure 2C–2F). Both MC38 and B16F10 cells express high levels of N-cadherin (Supplementary Figure 2). Primary tumor growth was inhibited by anti-KLRG1 + anti-PD-1 antibody greater than anti-KLRG1 antibody or anti-PD-1 antibody or control antibody alone (MC38: day 16 tumor volume 582 vs. 1259 vs. 1279 vs. 1461 mm^3^, respectively, Figure 2C; B16F10: day 21 tumor volume 544 vs 1799 vs 1464 vs 1687 mm^3^, respectively, Figure 2E). Anti-KLRG1 and anti-PD-1 antibody monotherapies had similar efficacy not significantly different from control antibody. Tumor growth inhibition by combination therapy was significantly greater than anti-PD-1 monotherapy (MC38 p=0.01; B16F10 p=0.007) or control antibody treatment (MC38 p=0.002; B16F10 p=0.0001) alone. Combination therapy resulted in a survival benefit compared to anti-PD-1 monotherapy (MC38 p=0.02 and B16F10 p=0.002) or compared to control antibody (MC38 p=0.008 and B16F10 p<0.0001) (Figures 2D, 2F). A tumor-free durable response (alive with complete regression of visible tumor at Day 35) was seen in 10% of combination therapy treated mice and 0% of all other cohorts in both models.

### KLRG1 expression is upregulated in human tumor after treatment with therapies that result in T cell proliferation

**Figure 3:**
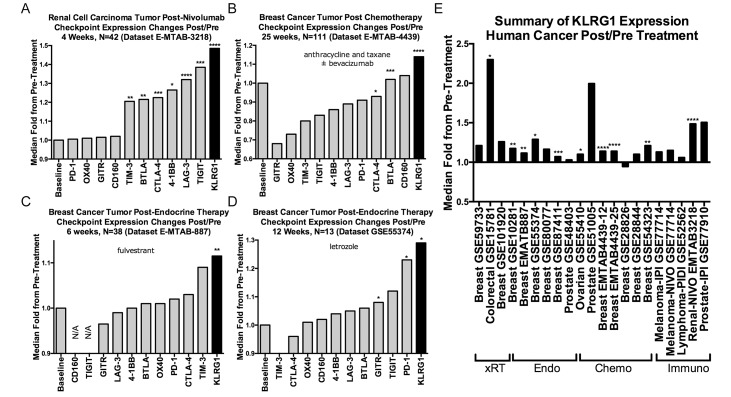
Effect of cancer treatments on KLRG1 and other checkpoint receptor expression in human cancer pre- and post-treatment tumor biopsy public domain datasets **(A–D)** Illustrative examples of checkpoint receptor changes compared to baseline pretreatment (fold-ratio = 1.0) after (A) immunotherapy, (B) chemotherapy, and (C, D). **(E)** Numerically increased post-treatment KLRG1 expression changes in 20/21 datasets organized by treatment modality. Abbreviations: xRT, radiation therapy; Endo, endocrine therapy; Chemo, chemotherapy; Immuno, immunotherapy. P-values: blank = not-significant; ^*^ < 0.05, ^**^<0.01, ^***^<0.001, ^****^<0.0001.

As KLRG1 expression increases as T cells differentiate in response to antigen stimulation [17], and a variety of cancer therapies are predicted to increase T cell differentiation, either directly (e.g., immunotherapy) or indirectly (e.g., immune activation after tumor cell death in chemotherapy and radiation therapy), we searched for all publicly available gene expression data from paired pre- and post-treatment human tumor samples, identifying 21 datasets (Supplementary Table 2). KLRG1 expression was numerically increased post-treatment in 20/21 (95%) of datasets, statistically significant in 10/21 (48%), in response to a range of treatments including radiotherapy, chemotherapy, endocrine therapy, and immunotherapies (including ipilimumab, nivolumab, and pidilizumab), over periods of time ranging from 1-25 weeks (Figure 3A–3E).

## DISCUSSION

Extensive preclinical murine cancer models and clinical development efforts have been undertaken for a number of co-inhibitory, or immune checkpoint, receptors that have been characterized as identifying exhausted T cells, including CTLA-4, PD-1, LAG-3, TIM-3, and TIGIT [3–5]. In contrast, the co-inhibitory receptor KLRG1 has been viewed as a marker of senescent T cells [10–12]. Very little human cancer data examining KLRG1 expression has been published, and no murine cancer models involving neutralization of KLRG1 have been reported.

Here, we illustrate through analyses of public domain data that KLRG1 marks a highly cytotoxic population of CD8 T and NK cells. Within the CD8 T cell population, KLRG1 expression is tied to antigen experience and differentiation status, an observation that has been previously emphasized [18], and aligned with cytotoxic potential, so that KLRG1 marks cells with the greatest cytotoxic potential. Although KLRG1 has infrequently been studied among TILS [18–21], single cell RNA-seq data indicates abundant KLRG1-expressing TILS across a range of cancer types, and significant numbers (13–26%) of CD8 T cells that do not express PD-1 but do express KLRG1.

Although KLRG1 signaling involves intracellular immunoreceptor tyrosine-based inhibitory motif (ITIM) domains and inhibition of Akt phosphorylation [15] resulting *in vitro* in T and NK cell inhibition, the *in vivo* function of KLRG1 has been unknown. The current *in vivo* KLRG1 neutralization studies confirm that KLRG1 restrains the immune system from cancer defense.

In the MC38 colon cancer and B16F10 melanoma models, monotherapy with KLRG1 was not significantly different than anti-PD-1 therapy, but combination therapy showed significant efficacy, including 10% of mice showing tumor regression and durable cure. Because cancer checkpoint immunotherapies induce T cell proliferation, they are predicted to expand the population of KLRG1+ cells, resulting in a homeostatic checkpoint brake on efficacy contributing to adaptive resistance. In animal models, this expansion of KLRG1+ cells has been observed with anti-CTLA-4 and anti-PD-1 [22], anti-4-1BB [23, 24], and HPV vaccine [25] therapies. The increase in KLRG1+ CD8 T cells with anti-PD-1 therapy, a phenomenon also seen in people treated with anti-PD-1 therapy, perhaps contributed to limited efficacy of anti-PD-1 monotherapy treatment in the MC38 model. Further studies using CD8 and NK cell depleted mice could elucidate the mechanism of anti-KLRG1 and anti-PD-1 combination efficacy.

More generally, here we have examined publicly available human cancer gene expression datasets and found that across a wide range of human tumors and therapies, including radiation, endocrine therapy, chemotherapy, and immunotherapy, KLRG1 is upregulated in post-treatment compared to pre-treatment tumor biopsies. These data suggest that the upregulation of the inhibitory checkpoint receptor KLRG1 could contribute to limited efficacy and adaptive resistance that develops with current immunotherapies [26], and suggests KLRG1 blockade may work efficaciously as a neo-adjuvant therapy.

Lastly, *in vitro* studies have demonstrated that KLRG1 is less inhibitory in mice than in humans, due to different amino acids at position 62 of its stalk region resulting in formation of KLRG1 monomers and oligomers in mice, but only dimers in humans [27]. This observation suggests that the inhibitory role of human KLRG1 in restraining anti-tumor responses is underestimated in mouse studies, and that anti-KLRG1 neutralizing antibody therapy could demonstrate substantial efficacy in people.

## MATERIALS AND METHODS

### Genomic datasets

All identified published flow cytometry data of CTLA-4, PD-1, and KLRG1 expression in human normal blood was compiled from publication tables and figures after literature searches were used to attempt to identify all available data sources (Supplementary Table 1). Bulk blood and tumor gene expression datasets were searched for KLRG1 and other relevant gene expression data, identifying data from the European Bioinformatics Institute ArrayExpress and the National Institutes of Health Gene Expression Omnibus (GEO) databases (Supplementary Table 2, 3). Median fold-ratios to baseline were used and testing of significance performed for unpaired (Mann–Whitney test) or paired (Wilcoxon matched pairs signed rank test) data using statistical software (Graphpad Prism). To understand immune checkpoint receptor expression at a single cell level in CD8+ T cells, human tumor single cell RNA-seq data from experiments with sufficient numbers of CD8+ T cells were identified. To understand KLRG1 (E- and N-cadherin) and PD-1 (PD-L1) ligand expression at a single cell level in cancer cells, human tumor single cell RNA-seq data that include malignant cell analysis were identified. For a global bulk tumor ligand expression view, the Cancer Genome Atlas (TCGA) data were downloaded from the Genomic Data Commons Data Portal (https://portal.gdc.cancer.gov) where gene expression levels were quantified with transcripts per million (TPM) using RSEM (http://deweylab.biostat.wisc.edu/rsem).

### Antibodies

Antibodies used for *in vivo* studies were anti-KLRG1 (ABC-m01, Abcuro, Inc.), anti-PD-1 (clone RMP1-14, BioXcell), and control (rat IgG2a, clone 2A3, BioXcell). Antibodies used for flow cytometry were anti-CD8a (clone 53-6.7, PerCP-Cy5.5, Beckton Dickinson) and anti-KLRG1 (clone 2F1, Biolegend).

### 4T1, MC38, and B16F10 models

For the 4T1 model, 20 female 6–8 week old BALB/c mice were inoculated subcutaneously with 1×10^5^ 4T1 cells suspended in 50μL RPMI 1640. Ten animals each were assigned into either of 2 groups, anti-KLRG1 or control antibody 10 mg/kg intraperitoneal injections on Days 1,4,7, and 11. For the MC38 and B16F10 models, 80 female 6–8 week old C57BL/6 mice were inoculated subcutaneously with 1×10^6^ MC38 cells (N=40 mice) or 1×10^6^ B16F10 cells (N=40 mice) suspended in 100μL DMEM. In each tumor model, ten animals each were assigned into 4 groups, control antibody (10 mg/kg), anti-KLRG1 (10 mg/kg), anti-PD-1 (5 mg/kg) (suboptimal dosage chosen to detect a combination effect), or anti-KLRG1 + anti-PD-1 on Day 6, 9, 12, 16 in the MC38 model and Day 5, 8, 11, and 15 in the B16F10 model. Randomized block designs based upon body weight and order of inoculation were used. Animals were sacrificed within 1 hour of when examined and found to have tumor volume of at least 2000 mm^3^ and, for 4T1, lungs examined for metastases, and for the MC38 anti-PD-1 treated cohort, blood sampled for flow cytometry detection of KLRG1 expression using ABC-m01. Animals were monitored daily and for tumor growth measured 3 times per week.

## SUPPLEMENTARY MATERIALS FIGURES AND TABLES




