# Supplementary material for: Co-inhibitory T cell receptor KLRG1: human cancer expression and efficacy of neutralization in murine cancer models
Source: Oncotarget. 2019 Feb 15;10(14):1399–406. doi: 10.18632/oncotarget.26659 (PMC6402715; doi:10.18632/oncotarget.26659)
Supplement: Supplementary file 1 [file oncotarget-10-1399-s001.pdf]

Co-inhibitory T cell receptor KLRG1: human cancer expression and efficacy of neutralization in murine cancer models

SUPPLEMENTARY MATERIALS

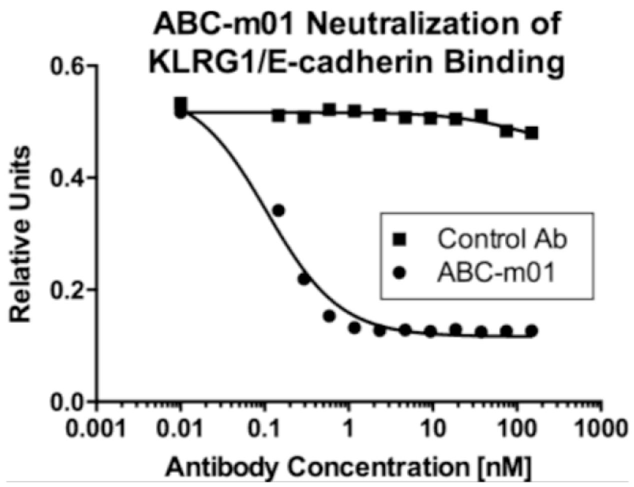

Supplementary Figure 1: Anti-KLRG1 antibody used in efficacy models neutralizes interaction of KLRG1 with E-cadherin in competition experiments.

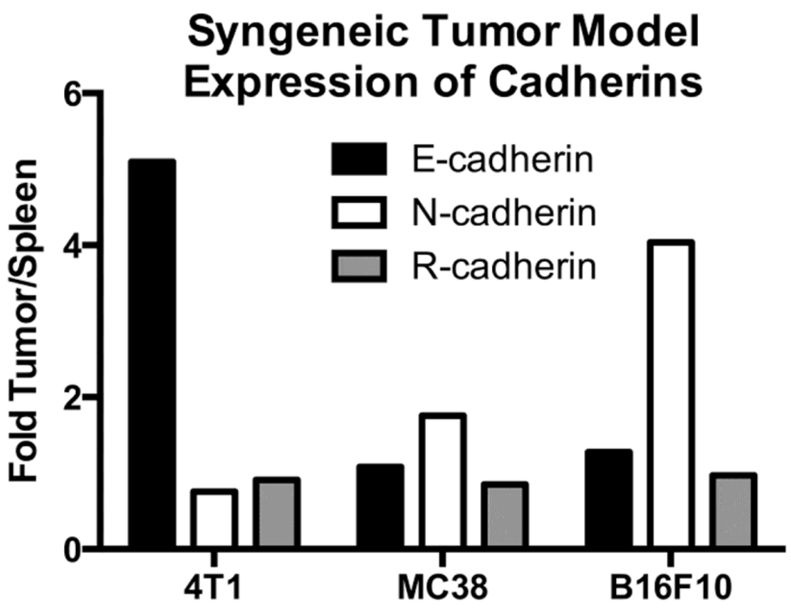

Supplementary Figure 2: Gene expression levels of KLRG1 ligands on 4T1, MC38, and B16F10 tumor samples. Data from Gene Expression Omnibus dataset GSE88509.

Supplementary Table 1: Healthy human blood % of indicated lymphocyte subsets that are CTLA-4+, PD-1+, or KLRG1+ by flow cytometry in published literature.

See Supplementary File 1

Supplementary Table 2: Single-cell RNAseq genomic datasets analyzed in Figure 2

Dataset	Cancer	Used for T Cell Analysis?	Used for Ligand Analysis?
GSE72046	Melanoma	Yes	Yes
GSE98638	HCC	Yes	No (T cells only)
GSE103322	HNSCC	Yes	Yes
GSE89567	Astrocytoma	Yes	No, cannot determine tumor cells
GSE75688	Breast cancer	No	Yes
GSE67980	Prostate CTC	No	Yes
GSE81861	CRC	No	Yes
GSE86978	Breast CTC	No	Yes

Supplementary Table 3: Genomic datasets analyzed in Figure 3

Dataset	Cancer	Treatment	Tissue Sample	Number of Patients	Duration of Treatment (Weeks)	KLRG1 Fold Ratio
GSE59733	Breast	Radiation	Tumor	9	1	1.21
GSE15781	Colorectal	Radiation	Tumor	9	10	2.30
GSE101920	Breast	Radiation	Tumor	5	?	1.33
GSE10281	Breast	Endocrine	Tumor	18	2	1.18
E-MTAB-887	Breast	Endocrine	Tumor	38	6	1.12
GSE55374	Breast	Endocrine	Tumor	13	12	1.29
GSE80077	Breast	Endocrine	Tumor	30	2 weeks	1.16
GSE87411	Breast	Endocrine	Tumor	109	2-4 weeks	1.07
GSE48403	Prostate	Endocrine	Tumor	7	22	1.03
GSE55410	Ovarian	Chemotherapy	Tumor	13	1	1.10
GSE51005	Prostate	Chemotherapy	Tumor	6	6	2.00
E-MTAB-4439-WK12	Breast	Chemotherapy	Tumor	97	12	1.14
E-MTAB-4439-WK25	Breast	Chemotherapy	Tumor	111	25	1.14
GSE28826	Breast	Chemotherapy	Tumor	14	12	0.94
GSE28844	Breast	Chemotherapy	Tumor	28	6-24	1.09
GSE54323	Breast	Chemotherapy	Tumor	14	2	1.21
GSE77714 ipi	Melanoma	Ipilimumab	Blood T cells	5	3	1.13
GSE77714 nivo	Melanoma	Nivolumab	Blood T cells	7	3	1.15
GSE52562	Lymphoma	Pidilizumab+rituximab	Tumor	8	16	1.06
E-MTAB-3218	Renal cell	Nivolumab	Tumor	42	4	1.49
GSE77910	Prostate	Ipilimumab	Tumor	6	Not specified	1.56
